# Audit of antenatal screening for syphilis and HIV in migrant and refugee women on the Thai-Myanmar border: a descriptive study

**DOI:** 10.12688/f1000research.4190.2

**Published:** 2015-05-15

**Authors:** Rose McGready, Joy Kang, Isabella Watts, Mary Ellen G Tyrosvoutis, Miriam B. Torchinsky, Aung Myo Htut, Nay Win Tun, Lily Keereecharoen, Chirapat Wangsing, Borimas Hanboonkunupakarn, François H. Nosten

**Affiliations:** 1 Shoklo Malaria Research Unit, 63110, Mahidol-Oxford Tropical Medicine Research Unit, Faculty of Tropical Medicine, Mahidol University, Mae Sot, 63110, Thailand; 2 Mahidol-Oxford Tropical Medicine Research Unit, Faculty of Tropical Medicine, Mahidol University, Bangkok, 10400, Thailand; 3 Centre for Tropical Medicine, Nuffield Department of Medicine, University of Oxford, Oxford, OX3 7BN, UK

**Keywords:** HIV syphilis pregnancy yaws refugees migrants Myanmar

## Abstract

Objective: The antenatal prevalence of syphilis and HIV/AIDS in migrants and refugees is poorly documented. The aim of this study was to audit the first year of routine syphilis screening in the same population and reassess the trends in HIV rates.

Methods: From August 2012 to July 2013, 3600 pregnant women were screened for HIV (ELISA) and syphilis (VDRL with TPHA confirmation) at clinics along the Thai-Myanmar border.

Results: Seroprevalence for HIV 0.47% (95% CI 0.30-0.76) (17/3,599), and syphilis 0.39% (95% CI 0.23-0.65) (14/3,592), were low. Syphilis was significantly lower in refugees (0.07% 95% CI 0.01-0.38) (1/1,469), than in migrants (0.61% 95% CI 0.36-1.04) (13/2,123). The three active (VDRL≥1:8 and TPHA reactive) syphilis cases with VDRL titres of 1:32 were easy to counsel and treat. Women with low VDRL titres (>75% were < 1:8) and TPHA reactive results, in the absence of symptoms and both the woman and her husband having only one sexual partner in their lifetime, and the inability to determine the true cause of the positive results presented ethical difficulties for counsellors.

Conclusion: As HIV and syphilis testing becomes available in more and more settings, the potential impact of false positive results should be considered, especially in populations with low prevalence for these diseases. This uncertainty must be considered in order to counsel patients and partners accurately and safely about the results of these tests, without exposing women to increased risk for abuse or abandonment. Our findings highlight the complexities of counselling patients about these tests and the global need for more conclusive syphilis testing strategies.

## Introduction

The global health impact of sexually transmitted infections (STIs) including HIV/AIDS and syphilis is well recognized ^1^. Both syphilis and HIV/AIDS pose major health risks in the developing world, impacting maternal and infant health due to vertical transmission via congenital infection and/or through breastfeeding ^2^. This is estimated to cause over 500,000 adverse pregnancy outcomes per year, including stillbirth and congenital infection ^1^. Syphilis more than any other bacterial and curable sexually transmitted infection, has greater potential to cause adverse birth outcomes when the diagnosis is missed ^3^. This was demonstrated in an area with a high rate of syphilis (7.7% amongst 19,878 women screened by RPR testing at antenatal clinic (ANC)) where, 51% of stillbirths, 24% of preterm live births, and 17% of all adverse pregnancy outcomes in unscreened women were attributable to maternal syphilis ^4, 5^. For the mother, untreated syphilis and HIV can cause multiple medical problems including death, and the open sores of syphilis increase the risk of human immunodeficiency virus (HIV) infection.

Displaced populations of migrants and refugees within developing regions are particularly vulnerable to disease although data on the prevalence of infection is scarce ^6^.

The Tak province on the Thai-Myanmar border is home to a diverse population comprised of local Thai and members of Thailand’s ethnic minorities as well as foreign migrant workers and refugees of multiple ethnicities from Myanmar ^7^. Members of the Karen ethnic group represent a large proportion of the ethnic minority people from both countries. The estimated 2 million displaced Burmese living in Thailand ^6^ are vulnerable to STIs and HIV/AIDs ^8^, due to the lack of access to health services, poor education and low income ^6, 8, 9^. In a cross-sectional study conducted amongst female sex-workers in 2009 in the major cities of Myanmar 18.4% of 136 participants tested positive for HIV and economic reasons were recognized as compelling to not use condoms with clients ^10^.

In 2005, we reported on cross sectional surveys of HIV and syphilis in pregnant refugee and migrant women from the Thai-Myanmar border which showed low rates of HIV (0.4%) and syphilis (0.4%) ^11^. While HIV screening in pregnancy has been routine since the cross sectional surveys syphilis screening was only introduced when funding became available. The aim of this study was to audit the first year of routine syphilis screening in the same population and reassess the trends in HIV rates.

## Methods

### Study site and population

The Shoklo Malaria Research Unit (SMRU) provides health services and conducts research of relevance to the local population of migrants and refugees on the border between Thailand and Myanmar ( www.shoklo-unit.com). As part of the efforts to reduce malaria-related maternal mortality ^12^, pregnant women are encouraged to attend SMRU antenatal clinics (ANC) as frequently as every fortnight. Indeed low attendance, or fewer than four antenatal visits (equivalent to the number of antenatal malaria screens) is a risk factor for maternal mortality in this setting ^12^. For refugees the service has been available since 1986, and for migrant communities, since 1998. Three SMRU clinics operate in border communities north and south of the town of Mae Sot: Maela (MLA,17°07′44″N 98°22′50″E) refugee camp (population circa 49,626) and in the migrant villages of Wang Pa (WPA,16°49′42″N 98°32′25″E) and Mawker Tai (MKT, 16°19′37″N 98°40′12″E) (population circa 42,000) ^13^. A recent publication using the WHO Safe Motherhood Needs Assessment of the quality of care at the facility recognized that the essential elements of ANC were all provided except syphilis testing ^14^.

These antenatal clinics are well attended and in the only formal survey undertaken more than 90% of women in refugee camps were found to be attending SMRU ANC ^15^. For migrants, a more mobile population, based on village population lists (42,000) and with no reason to believe birth rates would differ in migrants and refugees we suspect that ANC is equally well attended by this population ^13^. Antenatal clinic attendance and births in SMRU centres continues to increase yearly in the migrant population (WPA and MKT). Women are accepted anytime including at the time of birth, although the majority of women have already booked with the antenatal clinic. This audit was a non-selected inclusive cohort of all consecutively registered pregnant women in a one year period.

### Antenatal care

During the first antenatal visit, a dating ultrasound, routine screening blood tests (malaria smear, haematocrit, syphilis, HIV, full blood count) are taken, and medical and obstetric examinations are performed. Pre-test counselling, using an “opt-out” system, is provided to all women at their first antenatal visit before any screening blood tests are performed. Malaria smears are read promptly and positive cases are treated immediately. At all visits tablets of ferrous sulphate (200 mg daily), folic acid (5 mg weekly) and thiamine (vitamin B1 100 mg daily) ^16^ are supplied to all pregnant women. Anaemic patients receive 800 mg of ferrous sulfate and 5 mg of folic acid daily, and a tetanus vaccination is given to women who have not been previously immunized. The SMRU ANC program aims to provide integrated antenatal care for any medical or obstetric problem including treatment for HIV [life-long antiretroviral (ARV) triple therapy] or syphilis. ARV therapy was GPO-vir ^®^ (a combination of Stavudine (D4T) 30 mg, Lamivudine (3TC) 150 mg and Nevirapine (NVP) 200 mg) one tablet twice daily, for patients with low CD4 (<350/mm ^3^) counts; and for late pregnancy presentation (34 weeks of more) or CD4 (≥350/mm ^3^) then Zidovudine (AZT) 300 mg and Lamivudine (3TC) 150 mg as a combination tablet (ZilarVir) and Efavirenz (EFV) 600 mg taken in once dose once daily, is provided. Drug therapy for syphilis was benzathine penicillin G 2.4 million units by intramuscular injection.

### Laboratory sampling

Point of care HIV testing is done using an on-site rapid diagnostic test (Core™ HIV 1&2, Core Diagnostics, UK). The HIV rapid detection test (RDT) was carried out by SMRU laboratory technicians whose practice is standardized by regular quality control across the 3 SMRU sites by one of the authors (LK), and who have significant expertise on use of RDTs in malaria ^17^. At the first antenatal visit the results of the screening test are explained to the patient and the sera of positive RDT cases is transported to Mae Sot Hospital laboratory (30–60 km from the sites) for confirmation using an immunoassay (HIV Combi PT, Cobas ^®^, Roche, Germany). Mae Sot Hospital is the main Government Hospital for the Province of Tak and their practice is certified by Thailand Department of Public Health. Post-test counselling explaining the results of the confirmation test is provided the following week.

Syphilis testing is conducted at Mae Sot Hospital on a fee for service basis, on samples taken at SMRU ANCs. The hospital’s protocol ^18^ uses the Venereal Disease Research Laboratory (VDRL) test (VDRL Carbon Particle Antigen Kit, Plasmatec, Lab21 part of Health Care Ltd, UK) and confirms positive VDRL results with *Treponema pallidum* haemagglutination (TPHA) assay (TPHA kit, Plasmatec, Lab21 part of Health Care Ltd, UK). If a screening using VDRL is negative, no further tests are performed. Counselling about the test results is provided by SMRU staff to all women at their next antenatal visit. A policy to treat all patients for whom both VDRL and TPHA were reactive with 2.4 million units penicillin IM weekly × 3 doses was employed. This simple regimen which should be effective for all stages of the disease and prevention of congenital syphilis was used due to the difficulties in determining the stage of infection in most of our patients who denied symptoms or exposure history. No further serological testing was carried out after treatment in line with current recommendations for resource-poor settings ^19^.

### Factors associated with serological positive syphilis and HIV

Factors possibly associated with serological positive syphilis and HIV infection were examined by univariate and multivariate analysis ( Table 3). The small number of cases makes the confidence intervals on univariate and multivariate analysis wide. The serological positive syphilis risk factor were examined in detail to determine if a risk factor based screening would be possible (see supplementary materials).

### Ethical approval

Retrospective review of anonymized data from antenatal records was approved by the local Tak Community Advisory Board and the Oxford Tropical Research Ethics Committee (OXTREC 28-09).

### Statistical analysis

Data were analysed using SPSS for Windows™ (Version 20, SPSS Inc.) ( Dataset 1). Continuous normally distributed data were described by their means and compared with the Students’s *t* test, while non-normally distributed data were described by their median and compared with the Mann-Whitney U test. Percentages were calculated for categorical data, which were compared using the χ² test or Fisher’s exact test. Factors associated with a positive syphilis status or a positive HIV status, were compared by univariate analysis and odds ratios (OR) were calculated with a 95% confidence interval. Factors associated with a diagnosis of syphilis, and a diagnosis of HIV, were evaluated by univariate analysis; two logistic regression models were created using “syphilis (yes/no)” and “HIV (yes/no)” as dependent variables. All factors with a P value <0.10 in univariate analysis were entered in their respective stepwise forward logistic regression model, and were included in the relevant tables. Adjusted odds ratios (AOR) were given with their 95% confidence interval.

HIV and syphilis antenatal screening data at SMRU 2012-13 The file is a SAV type file with all variables used in the analysis available in the dataset (syp_hivF1000.sav) Click here for additional data file. Copyright: © 2015 McGready R et al. 2015 Data associated with the article are available under the terms of the Creative Commons Zero "No rights reserved" data waiver (CC0 1.0 Public domain dedication).

## Results

From the 8 ^th^ of August 2012, until the 7 ^th^ of August 2013, there were 3,600 women who attended the SMRU antenatal clinics at least once. Most were regular attenders. The ethnic make-up of the refugee and migrant population was largely Karen and Burmese, and significant differences between baseline characteristics of refugee and migrant clinics and between the two migrant clinics were apparent ( Table 1). Maela has the highest case load (1,475 ANC attenders), followed by Wang Pha (1,171) and then Maw Ker Thai (954). Age and gravidity were similarly matched at all clinics, but women attending the migrant sites (WPA and MKT) had a significantly higher number of remarriages and a shorter duration of residence at their current address when compared with MLA. Duration of residence and literacy (self-reported ability to read) was lowest in WPA. In this border population, country of residence differed significantly between sites, with only 8% of ANC patients in MLA reporting an address in Myanmar, compared with 34% in MKT and 67% in WPA. Finally, significant differences were seen in the ethnic makeup of the populations. Karen ethnicities account for 82% of MLA patients, but only around 30% of the patients in MKT and WPA. Burmese Muslim patients make up 12% of MLA’s ANC attenders, but are less than 1% of the migrant populations. Around 40% of the migrant patients are ethnically Burman, but less than 2% of the MLA patients report Burman ethnicity. Other ethnic minorities comprise 10% of MKT’s patients, 5% of WPA and only 2% of MLA.

**Table 1.** Baseline characteristics of pregnant refugees and migrants enrolled to SMRU antenatal clinics.

	Refugee	Migrant	
Characteristics	Maela N=1,475	Maw Ker Thai N=954	Wang Pha N=1,171
Age in years, mean ± standard deviation [range]	26.5±7 [14–48]	26±7 ^c^ [15–45]	27±7 [14–46]
Gravidity, median [range]	2 [1–14]	2 [1–12]	2 [1–12]
Primigravidae, % (n)	30.9 (456)	32.0 (305)	30.1 (352)
Median number marriages per woman [range]	1 [1–3] ^c, d^	1 [1–5]	1 [1–3]
Myanmar address, % (n)	8.1 (119) ^c, d^	33.9 (323) ^c^	66.8 (782)
Years at current address, median [range]	7 ^c, d^ [0–43]	3 ^c^ [0–42]	2 [0–40]
Literate, % (n)	64.1 (945) ^c^	64.8 (618) ^c^	51.1 (598)
Ethnic group ^a^			
Sgaw Karen	73.1 (1078)	29.7 (283)	29.1 (341)
Mixed Karen (Sgaw and Poe)	2.2 (32)	3.7 (35)	2.9 (34)
Poe Karen	7.5 (111)	13.6 (130)	18.5 (217)
Burman Muslim	11.9 (175)	0.4 (4)	0.2 (2)
Burman	1.8 (27)	38.3 (365)	40.6 (476)
Mixed Karen and Burman Muslim/Burman/Other	1.5 (23)	3.9 (38)	3.5 (42)
Other ^b^ Ethnic group e.g. Mon, Pa-Oh, Rakhine, Shan, Chin	2.0 (29)	10.2 (97)	4.9 (57)
Burman and Burman Muslim	0	0.2 (2)	0.2 (2)

^a^Ethnic group of the woman was derived from the ethnicity of the woman’s parents. Sgaw Karen implies both parents were Sgaw Karen and so on. Burman Muslim is used locally to define people who originated from Bangladesh or Rhakine state. ^b^Other as in not one of the leading 3 ethnic groups (Karen, Burman or Burman Muslim) ^c^P<0.05 significantly different Wang Pha; ^d^P<0.05 significantly different Maw Ker Thai;

### Syphilis

Syphilis was tested in 3,592 of 3,600 women (99.78%). The remaining 8 patients were not tested due to interruption of the usual screening process such as a patient actively miscarrying. Off-site testing found 0.50% (18/3,592) VDRL reactive of whom 22.2% (4/18) were TPHA non-reactive indicating biological false positive reactions. Prevalence of serological syphilis (VDRL and TPHA reactive sera) was 0.39% (95% CI 0.23–0.65) (14/3,592). Of these, the majority 78.6% (11/14) were low VDRL titres <1:8 (three were 1:2; eight were 1:4) and the remaining three were all 1:32. Only two women were symptomatic, both at a titre of 1:32 and one of these women was also HIV positive. The proportion of serological syphilis in MLA, MKT and WPA was 0.07% (1/1,469), 0.73% (7/954) and 0.51% (6/1,169) respectively. Syphilis prevalence was significantly lower in MLA compared to MKT P=0.008 and WPA P=0.049, but there was no difference between the two migrant sites (P=0.583). The overall prevalence of syphilis was lower in refugees 0.07% (1/1,469) (95% CI 0.01–0.38) compared to migrants 0.61% (13/2,123) (95% CI 0.36–1.04), P=0.011.

All active syphilis cases (n=3) found in this audit (titre ≥1:8 and TPHA reactive) were in young migrant women who were also primigravidae and all were treated. Amongst their partners, one partner agreed to testing and treatment (HIV and syphilis positive case); one agreed to treatment but not to testing (his wife had a titre of 1:32 and one marriage) and one was not contactable. Amongst the 11 low titre couples: five husbands attended the clinic and all five were negative (VDRL titres in their wives were 1:4 (four cases) and 1:2 (one case)); the remaining six husbands did not get tested because they were away for work (five cases) and one woman never returned at all after the first consultation. Counselling couples with low VDRL titres who both reported to have one lifetime sexual partner was particularly challenging.

Treatment with IM Penicillin was given to 71% (10/14) of patients with serological syphilis (positive VDRL and TPHA reactive) and four low titre (2 with 1:2 and 2 with 1:4) women remained untreated. Three of these women had low risk histories (no history of symptoms and reporting only one lifetime sexual partner for both the woman and her husband) and one history was unknown as the woman never returned to ANC after the first visit.

### HIV

A HIV test was performed on 3,599 of 3,600 women (99.9%) of whom 0.9% (34) were tested positive by a single on-site rapid diagnostic test (RDT). Off-site confirmation testing by double ELISA showed that 46.9% (95% CI 30.9–63.6%) (17/32) of these positive RDT results were false positives (including 2 cases for whom confirmation testing was initially indeterminate, but were ultimately negative on repeat samples). This high rate of RDT biological false positives is not unexpected in a low transmission setting as these tests are optimized for sensitivity at the expense of specificity ^20^. The confirmed HIV-positive rate in pregnancy was 0.47% (95% CI 0.30–0.76) (17/3,599). Lowest HIV rates were again observed in the refugee camp MLA 0.27% (4/1,474) compared to the migrant sites, MKT 0.52% (5/594) and WPA 0.68% (8/1,171). While MLA was significantly lower than WPA (P=0.049) no other significant differences were observed: MLA vs. MKT P=0.329, and MKT vs. WPA P=0.783. The percentage of HIV cases in refugees was not significantly different from the combined percentage of the two migrant sites: 0.3% (95% CI 0.11–0.70) (4/1,474) vs. 0.61% (13/2125) (95% CI 0.36–1.0), P=0.215. There were 82.4% (14/17) of HIV-positive women treated with ARVs following guidelines based on WHO recommendations. The three untreated women were all migrants: one decided to return to Myanmar; one miscarried with a very high CD4 count and was followed up with 6-monthly CD4 counts and one woman did not return for the result.

### Trends over time

There has been no significant change in the prevalence of HIV and syphilis from 1997 ^7^ to 2013 in refugees, nor for syphilis ^7^ in migrants 2005 to 2013. While data from Thailand ^21^ is reported for comparison we were unable to obtain the raw data for confidence interval analysis ( Table 2). For Myanmar ^22^ it was possible to obtain raw data for the whole country combined and by location, so Myawaddy data, from the Burmese town opposite Mae Sot, Thailand, was included. The data were collected from 1st Mar–31st May 2012 from 35 sentinel sites with a projected sample size of 400 from each site. Of note is the higher rate in Myawaddy than all Myanmar combined for HIV and syphilis. A comment from the report: “syphilis prevalence was highest among pregnant women in age group 40–44 as 0.6% (4/492)” was similar to observations in the SMRU data.

**Table 2.** Trends in antenatal HIV and syphilis prevalence (95% CI) in migrant and refugee women at SMRU from surrounding areas of Thailand and Myanmar.

	Population	1997 Cross-sectional	2005 Cross-sectional	Aug-2012–Jul-2013 Population cohort
Syphilis	Refugee ^a, b^	0 (0–0.9)% (0/404)	0.40 (0.1–1.2)% (3/741)	0.07 (0.01–0.38)% (1/1,469)
	Migrant ^a, b^	n.a	0 (0–1.6) (0/234)	0.61 (0.36–1.04)% (13/2,123)
	Thailand ^c^	n.a	0.13%	0.1%
	Myanmar ^c^	n.a.	2.0%	0.32 (0.24–0.43)% (45/13,995)
	Myawaddy ^c^			0.50 (0.14–0.18)% (2/400)
HIV	Refugee ^b^	0.2 (0–1.1)% (0/500)	0.40 (0.1–1.4)% (2/500)	0.27 (0.11–0.70)% (4/1,474)
	Migrant ^b^	n.a	n.a.	0.61 (0.36–1.04)% (13/2,125)
	Thailand ^c^	1.75%	0.86%	0.59%
	Myanmar ^c^	1.5%	1.3%	0.80 (0.67–0.96)% (112/13995)
	Myawaddy ^c^			1.5 (0.39–2.54)% (4/400)

^a^Serological syphilis positive using the same criteria, and the same hospital for confirmatory testing at each survey time point; n.a. not available ^b^Data from refugee and migrant populations in 1997 and 2005; published in reference ^7^ ^c^Data for Thailand from reference ^21^ and Myanmar from reference ^22^

Factors possibly associated with serological positive syphilis and HIV infection were examined by univariate and multivariate analysis ( Table 3). The small number of cases makes the confidence intervals on univariate and multivariate analysis wide. On multivariate analysis, older maternal age and a short history of residence at the current address were risk factors for syphilis; and remarriage and non-Karen parentage were risk factors for HIV; however more data is required to confirm this.

**Table 3.** Factors associated with antenatal syphilis and HIV, Thai-Myanmar border.

Variable		Syphilis ^a^				HIV ^a^			
		N	% (n)	OR (95% CI) P value	AOR (95% CI) P value	N	% (n)	OR (95% CI) P value	AOR (95% CI) P value
Group	Refugee	1,469	0.1 (1)	Reference	Reference	1,474	0.3 (4)	Reference	Reference
	Migrant	2,123	0.6 (13)	**9.045 (1.182–69.214)** **P=0.034**	3.461 (0.407–29.454) P=0.256	2,125	0.6 (13)	2.262 (0.736–6.951) P=0.15	Not included
Marriage	Only 1	2,813	0.2 (7)	Reference	Reference	2,819	0.2 (6)	Reference	Reference
	>1	779	0.9 (7)	**3.635 (1.271–10.394)** **P=0.016**	2.507 (0.851–7.391) P=0.096	780	1.4 (11)	**6.706 (2.472–18.192)** **P<0.001**	**5.497 (1.982–15.247)** **P<0.001**
Parity	Primipara	1,112	0.4 (4)	Reference	Reference	1,113	0.3 (3)	Reference	Reference
	Multipara	2,480	0.4 (10)	0.122 (0.351–3.583) P=0.847	Not included	2,486	0.6 (14)	2.095 (0.601–7.306) P=0.246	Not included
Literacy (self-reported)	Literate	2,158	0.3 (7)	Reference	Reference	1,439	0.4 (6)	Reference	Reference
	Illiterate	1,434	0.5 (7)	1.507 (0.528–4.307) P=0.444	Not included	2,160	0.5 (11)	0.818 (0.302–2.217) P=0.693	Not included
Age	<30 y	2,408	0.2 (4)	Reference	Reference	2,412	0.3 (7)	Reference	Reference
	≥30 y	1,184	0.8 (10)	**5.119 (1.602–16.357)** **P=0.006**	**4.632 (1.417–15.139)** **P=0.011**	1,187	0.8 (10)	**2.919 (1.108–7.688)** **P=0.030**	2.094 (0.0777–5.648) P=0.144
Parents	At least 1 Karen	2,386	0.1 (3)	Reference	Reference	2,392	0.2 (5)	Reference	Reference
	Neither Karen	1,206	0.9 (11)	**7.312 (2.036–26.258)** **P=0.002**	3.799 (0.987–14.681) P=0.052	1,207	1.0 (12)	**4.794 (1.685–13.639)** **P=0.003**	**4.514 (1.581–12.887)** **P=0.005**
Residence	Thailand	2,370	0.3 (8)	Reference	Reference	2,376	0.4 (10)	Reference	Reference
	Myanmar	1,222	0.5 (6)	1.457 (0.504–4.208) P=0.487	Not included	1,223	0.6 (7)	1.362 (0.517–3.587) P=0.532	Not included
Length residence	≥6mths	2,965	0.2 (6)	Reference	Reference	2,972	0.5 (14)	Reference	Reference
	<6mths	627	1.3 (8)	**6.374 (2.204–18.434)** **P=0.001**	**4.220 (1.471–13.297)** **P=0.008**	627	0.5 (3)	1.016 (0.291–3.545) P=1.000	Not included

OR=Odds ratio; AOR=adjusted odds ratio

^a^1 woman had both HIV and syphilis; including HIV in the syphilis model; or syphilis in the HIV model did not change the AOR values

## Conclusions

There is limited research on the prevalence of HIV and syphilis in migrant and refugee pregnant populations, despite the vulnerability of these populations. Here we describe low and stable HIV and syphilis prevalence in refugees over a 15–16 year period, on the Thai-Myanmar border. This is supported by low published rates in refugees from the same area resettled in new countries ^23^. The data for migrants is newly available and while no statistically significant difference in HIV rates was observed between refugees and the combined migrant population, HIV rates were higher in the migrant group situated closer to the Mae Sot and Myawaddy townships. Syphilis was almost absent in the refugees and prevalence in the migrants on a par with data from Myanmar ^22^ and comparable to regional and world averages ^1^. The data presented here are contextual and are not presented to detract from the vulnerability of refugee and migrant women who are at increased risk of HIV and syphilis infection, due to transient marital relationships based on personal security or unwanted sexual attacks ^24^. Rather, to question the prevalence at which routine screening of all pregnant women for HIV and, in particular, syphilis is no longer advisable ^25^ and to describe the situation from a low prevalence, low resource setting. In addition and for clarity for the reader, while the distinction between refugee and migrant women is reasonable, publications from this border do not usually try to distinguish migrants from urban Mae Sot and those from the rural areas of Tak province ^26^. In this publication the MKT population is about 60 km from Mae Sot and the WPA population only 30 km from Mae Sot. Rural, predominantly agricultural workplaces are likely to present less risk from sexually transmitted infections than urban occupations including domestic and services work and factory jobs. It would be useful to make a more formal comparison of these risks in the future as these could affect the recommendations for antenatal screening.

Very little confidence can be placed in the multivariate analysis and it is questionable whether it should be presented at all given the low number of cases and subsequently wide confidence intervals. Somewhat reassuring in terms of data robustness is the association of HIV with the number of remarriages, similar to the number of partners in other settings, and a risk factor typically described in HIV epidemiology ^27^. There is an association suggesting protection from HIV with Karen parentage. Karen culture, which does allow for remarriage in cases of divorce or death of a spouse, holds a strong taboo on multiple sexual partners or extramarital sex. This taboo may provide some protection in the Karen-dominated populations in the refugee camp and is supported by the ethnic trend shown in Table 1 and Table 3.

Older age of women as a risk factor for syphilis is consistent with the Myanmar National AIDS Programme report ^22^. This is also suggestive of another confounder to our analysis, the fact that existing serologic tests cannot differentiate between syphilis and yaws or other non-venereal treponematoses ^28, 29^. Yaws is still included in Thailand’s program of neglected tropical diseases indicating that total eradication may not have been achieved yet ^30^. The last reported outbreak of yaws in Thailand was published in 1994 (within our patients’ lifetime) and occurred in a remote village a few hundred kilometres south of the SMRU sites ^31^. The last yaws-related publication from Myanmar was published in 1960 ^32^ on the yaws national programme. Myanmar has been amongst the world’s poorest 30 countries for decades, and populations on the borders, remote from central government, have had poor access to health services ^31, 32^. If a national program has been implemented to eradicate Yaws, it is unlikely it has reached these communities. More than 75% of TPHA-reactive patients in our cohort had low VDRL titres <1:8 and the active cases were in all in younger women ^30^. Amongst these low titre women, all of the husbands who came to the clinic were negative and the couples presented low risk histories. Latent syphilis (acquired via sexual contacts not disclosed by the patients) cannot be ruled out but the picture is suggestive of an unidentified treponemal infection (such as yaws) causing false-positive results. Publically available data of VDRL titres in pregnant women in Thailand are similar to what was observed here and are in contrast to studies from Africa, where a greater proportion of high sera titres are reported ^5, 33^. Counselling VDRL discordant couples with low risk histories, where the woman has a very low VDRL titer presents an ethical challenge in this conservative culture. The potential for serious social consequences, such as abuse or abandonment, from false positive results in such couples should be considered.

While the stable HIV prevalence in this population supports continuation of routine HIV screening for all pregnant women, the cost-effectiveness of the syphilis screening in the refugee population is unclear and this would be an area of valuable future research. Donor funds for these screening programs remain precarious and increasingly difficult to sustain as highlighted by Spiegel *et al.*, ^34^ and as organizations realign services with funding constraints, it is the least cost-effective that will be the first to be abandoned. In low prevalence settings where routine syphilis screening is carried out, healthcare workers should be prepared to counsel low risk couples with discordant screening results, and such counselling should include an explanation of the possibility of false-positive results due to exposure to yaws or other non-venereal treponematoses in countries where the disease has been or remains endemic ^30^.

There are limitations with this data series. There are women, estimated at less than 10% of all pregnant women in the catchment area, who do not attend antenatal or delivery care, and obviously we cannot know their status to determine whether they are more or less at risk than women who attended antenatal care. In some viewpoints the lack of point of care (POC) tests, promoted by WHO as the way forward to eliminate maternal transmission of HIV and syphilis, would be considered a limitation ^35^. The experience at SMRU has only been with POC for HIV (higher positivity rate than syphilis) and their performance is not encouraging as nearly half were false positives. As the incidence goes down the performance of POC tests likewise falls. Currently maternal distress while waiting for confirmation of a positive POC test for HIV which is sent to the local Thailand Government hospital is a minimum of 3 days. Syphilis, with a lower prevalence than HIV, is likely to have a higher rate of false positive POC tests and for this reason have not been introduced ^36^.

Reports from 2011 estimate over 7 million people live in protracted refugee situations, and over 27 million are internally displaced persons ^37^ and contextual differences within such groups are highlighted here where differences were found in quite similar populations. While these results cannot be widely applied to other settings, the questions raised about unintended consequences of routine screening and the need for more conclusive syphilis testing strategies, have implications with global relevance. The overall cost-effectiveness and impact of syphilis serological testing in pregnancy in low prevalence areas requires more in-depth evaluation especially in settings where funding for the most basic health care needs remains precarious.

## Data availability

The data referenced by this article are under copyright with the following copyright statement: Copyright: © 2015 McGready R et al.

Data associated with the article are available under the terms of the Creative Commons Zero "No rights reserved" data waiver (CC0 1.0 Public domain dedication). 

*figshare:* HIV and syphilis antenatal screening data at SMRU 2012–13. Doi: 10.6084/m9.figshare.1044120 ^38^

## Figures and Tables

**Table:** 

Score	Syphilis n=14	No syphilis n=3578
**0**	0 (0)	646 (100%)
**1**	2 * (0.2%)	1082 (99.8%)
**2**	0 (0)	1032 (0%)
**3**	4 (0.7%)	575 (99.3%)
**4**	5 (2.3%)	216 (97.7%)
**5**	3 (10.0%)	27 (90.0%)

*Score of one obtained in one case due to ‘migrant’ status, and in the other case because the woman was ≥30 years old.

**Table:** 

Risk factors	Status	Age	Remarriage ^*^	Ethnic group ^^^	Residence
**1**	Refugee	>=30	1	Karen	>=6mths
**1**	Migrant	<30	1	Karen	>=6mths
**3**	Migrant	<30	1	not Karen	<6mths
**3**	Migrant	>=30	1	not Karen	>=6mths
**3**	Migrant	>=30	1	not Karen	>=6mths
**3**	Migrant	>=30	>1	Karen	>=6mths
**4**	Migrant	<30	>1	not Karen	<6mths
**4**	Migrant	<30	>1	not Karen	<6mths
**4**	Migrant	>=30	1	not Karen	<6mths
**4**	Migrant	>=30	1	not Karen	<6mths
**4**	Migrant	>=30	>1	not Karen	>=6mths
**5**	Migrant	>=30	>1	not Karen	<6mths
**5**	Migrant	>=30	>1	not Karen	<6mths
**5**	Migrant	>=30	>1	not Karen	<6mths

^*^ *one of the partners with more than one marriage*

^^^ *Karen = at least one of the pregnant woman’s parents of Karen ethnicity*

## Supplementary materials

Each woman in the database was scored with a one for each of the following risk factors: migrant, age ≥30 years, history of remarriage, neither parent of the pregnant woman was of the Karen ethnic group and residence of <6 months at the current address, otherwise the woman was given a score of zero. The five risk factors were tallied for each woman. Most women with syphilis had a score of three or more and accounted for 1.45% (12/830) of all women with a score of three of more. Women with syphilis and a score of less than three included 0.07% (2/2762). Limiting screening to a risk factor score of 3 or more would include less than ¼ of women attending ANC 23% (830/3600), and omit approximately 14.3% (2/14) of cases.

Another attempt to identify risk factors was to identify a pattern of risk factors and this is shown in the table below.

**Conclusion**: there is insufficient data on which to base a protocol of risk factor based screening.

## References

[1] Newman L Kamb M Hawkes S: Global estimates of syphilis in pregnancy and associated adverse outcomes: analysis of multinational antenatal surveillance data. *PLoS Med.* 2013;10(2):e1001396. 10.1371/journal.pmed.1001396 23468598 PMC3582608

[2] Chaudhary M Kashyap B Bhalla P: Congenital syphilis, still a reality in 21 ^st^ century: a case report. *J Med Case Rep.* 2007;1:90. 10.1186/1752-1947-1-90 17877837 PMC2034583

[3] Chico RM Hack BB Newport MJ: On the pathway to better birth outcomes? A systematic review of azithromycin and curable sexually transmitted infections. *Expert Rev Anti Infect Ther.* 2013;11(12):1303–32. 10.1586/14787210.2013.851601 24191955 PMC3906303

[4] Watson-Jones D Changalucha J Gumodoka B: Syphilis in pregnancy in Tanzania. I. Impact of maternal syphilis on outcome of pregnancy. *J Infect Dis.* 2002;186(7):940–7. 10.1086/342952 12232834

[5] Watson-Jones D Gumodoka B Weiss H: Syphilis in pregnancy in Tanzania. II. The effectiveness of antenatal syphilis screening and single-dose benzathine penicillin treatment for the prevention of adverse pregnancy outcomes. *J Infect Dis.* 2002;186(7):948–57. 10.1086/342951 12232835

[6] Carballo M Nerukar A: Migration, refugees, and health risks. *Emerg Infect Dis.* 2001;7(3 Suppl):556–60. 10.3201/eid0707.017733 11485671 PMC2631841

[7] Kusakabe K Pearson R: Transborder Migration, Social Reproduction and Economic Development: A Case Study of Burmese Women Workers in Thailand. *Int Migr.* 2010;48(6):13–43. 10.1111/j.1468-2435.2010.00630.x

[8] Salama P Spiegel P Brennan R: No less vulnerable: the internally displaced in humanitarian emergencies. *Lancet.* 2001;357(9266):1430–1. 10.1016/S0140-6736(00)04570-0 11356464

[9] Spiegel PB: HIV/AIDS among conflict-affected and displaced populations: dispelling myths and taking action. *Disasters.* 2004;28(3):322–39. 10.1111/j.0361-3666.2004.00261.x 15344944

[10] Swe LA Rashid A: HIV prevalence among the female sex workers in major cities in Myanmar and the risk behaviors associated with it. *HIV AIDS (Auckl).* 2013;5:223–30. 10.2147/HIV.S50171 24039455 PMC3770521

[11] Plewes K Lee T Kajeechewa L: Low seroprevalence of HIV and syphilis in pregnant women in refugee camps on the Thai-Burma border. *Int J STD AIDS.* 2008;19(12):833–7. 10.1258/ijsa.2008.008034 19050214

[12] McGready R Boel M Rijken MJ: Effect of early detection and treatment on malaria related maternal mortality on the north-Western border of Thailand 1986–2010. *PLoS One.* 2012;7(7):e40244. 10.1371/journal.pone.0040244 22815732 PMC3399834

[13] Carrara VI Lwin KM Phyo AP: Malaria burden and artemisinin resistance in the mobile and migrant population on the Thai-Myanmar border, 1999–2011: an observational study. *PLoS Med.* 2013;10(3):e1001398. 10.1371/journal.pmed.1001398 23472056 PMC3589269

[14] Hoogenboom G Thwin MM Velink K: Quality of intrapartum care by skilled birth attendants in a refugee clinic on the Thai-Myanmar border: a survey using WHO Safe Motherhood Needs Assessment. *BMC Pregnancy Childbirth.* 2015;15(1):17. 10.1186/s12884-015-0444-0 25652646 PMC4332741

[15] Luxemburger C McGready R Kham A: Effects of malaria during pregnancy on infant mortality in an area of low malaria transmission. *Am J Epidemiol.* 2001;154(5):459–65. 10.1093/aje/154.5.459 11532788

[16] McGready R Simpson JA Cho T: Postpartum thiamine deficiency in a Karen displaced population. *Am J Clin Nutr.* 2001;74(6):808–13. 11722964 10.1093/ajcn/74.6.808

[17] Mens PF de Bes HM Sondo P: Direct blood PCR in combination with nucleic acid lateral flow immunoassay for detection of Plasmodium species in settings where malaria is endemic. *J Clin Microbiol.* 2012;50(11):3520–5. 10.1128/JCM.01426-12 22915610 PMC3486265

[18] Wiwanitkit V: Screening for syphilis in pregnancy: which is the proper method? *Arch Gynecol Obstet.* 2007;276(6):629–31. 10.1007/s00404-007-0400-y 17569069

[19] Saloojee H Velaphi S Goga Y: The prevention and management of congenital syphilis: an overview and recommendations. *Bull World Health Organ.* 2004;82(6):424–30. 15356934 PMC2622853

[20] Shanks L Klarkowski D O’Brien DP: False positive HIV diagnoses in resource limited settings: operational lessons learned for HIV programmes. *PLoS One.* 2013;8(3):e59906. 10.1371/journal.pone.0059906 23527284 PMC3603939

[21] Country Profile Data Sheets - Thailand.2013 Reference Source

[22] HIV Sentinel Sero-surveillance Survey Report, 2012. National AIDS Programme, Department of Health, Ministry of Health Myanmar [Internet].2013 Reference Source

[23] Chaves NJ Gibney KB Leder K: Screening practices for infectious diseases among Burmese refugees in Australia. *Emerg Infect Dis.* 2009;15(11):1769–72. 10.3201/eid1511.090777 19891864 PMC2857247

[24] Spiegel PB Mills E Joffres MR: The imperative to respond with appropriate and timely interventions to mass rape in conflict-affected areas. *AIDS.* 2011;25(3):391. 10.1097/QAD.0b013e3283420e7b 21239895

[25] Kahn JG Jiwani A Gomez GB: The cost and cost-effectiveness of scaling up screening and treatment of syphilis in pregnancy: a model. *PloS one.* 2014;9(1):e87510. 10.1371/journal.pone.0087510 24489931 PMC3906198

[26] Rattanapunya S Kuesap J Chaijaroenkul W: Prevalence of malaria and HIV coinfection and influence of HIV infection on malaria disease severity in population residing in malaria endemic area along the Thai-Myanmar border. *Acta tropica.* 2015;145:55–60. 10.1016/j.actatropica.2015.02.001 25728746

[27] Kainne Dukubo E Kim AA Le LV: HIV incidence in Asia: a review of available data and assessment of the epidemic. *AIDS Rev.* 2013;15:67–76. 23681434

[28] Noordhoek GT Cockayne A Schouls LM: A new attempt to distinguish serologically the subspecies of Treponema pallidum causing syphilis and yaws. *J Clin Microbiol.* 1990;28(7):1600–7. 2199521 10.1128/jcm.28.7.1600-1607.1990 PMC267996

[29] Noordhoek GT Wieles B van der Sluis JJ: Polymerase chain reaction and synthetic DNA probes: a means of distinguishing the causative agents of syphilis and yaws? *Infect Immun.* 1990;58(6):2011–3. 2187816 10.1128/iai.58.6.2011-2013.1990 PMC258761

[30] Narain JP Dash AP Parnell B: Elimination of neglected tropical diseases in the South-East Asia Region of the World Health Organization. *Bull World Health Organ.* 2010;88(3):206–10. 10.2471/BLT.09.072322 20428388 PMC2828791

[31] Tharmaphornpilas P Srivanichakorn S Phraesrisakul N: Recurrence of yaws outbreak in Thailand, 1990. *Southeast Asian J Trop Med Public Health.* 1994;25(1):152–6. 7825005

[32] Tha Hla U: The proposed national programme on the control of yaws in Burma. *Burma Med J.* 1960;8:96–7. 13776148

[33] Taiwo SS Adesiji YO Adekanle DA: Screening for syphilis during pregnancy in Nigeria: a practice that must continue. *Sex Transm Infect.* 2007;83(5):357–8. 10.1136/sti.2006.023416 17493980 PMC2659024

[34] Spiegel PB Hering H Paik E: Conflict-affected displaced persons need to benefit more from HIV and malaria national strategic plans and Global Fund grants. *Confl Health.* 2010;4:2. 10.1186/1752-1505-4-2 20205901 PMC2827465

[35] Jafari Y Peeling RW Shivkumar S: Are Treponema pallidum specific rapid and point-of-care tests for syphilis accurate enough for screening in resource limited settings? Evidence from a meta-analysis. *PLoS One.* 2013;8(2):e54695. 10.1371/journal.pone.0054695 23468842 PMC3582640

[36] Shahrook S Mori R Ochirbat T: Strategies of testing for syphilis during pregnancy. *Cochrane Database Syst Rev.* 2014;10:CD010385. 10.1002/14651858.CD010385.pub2 25352226 PMC11126892

[37] UNHCR. War's Human Cost. Geneva: UNHCR,2013 Reference Source

[38] McGready R Kang J Watts I: HIV and syphilis antenatal screening data at SMRU 2012–13. *figshare.* 2014 Data Source

