# Supplementary material for: Audit of antenatal screening for syphilis and HIV in migrant and refugee women on the Thai-Myanmar border: a descriptive study
Source: F1000Res. 2015 May 15;3:123. Originally published 2014 Jun 10. [Version 2] doi: 10.12688/f1000research.4190.2 (PMC4654433; doi:10.12688/f1000research.4190.2)
Supplement: Peer review history

# Peer review: Referee response for version 2

**DOI:** 10.5256/f1000research.6971.r10130

**Published:** 2015-08-26

**Authors:** Nitika Pant Pai

**Affiliations:** 1 Division of Clinical Epidemiology, Department of Medicine, Royal Victoria Hospital, McGill University Health Centre, Montreal, QC, Canada

I have read this submission. I believe that I have an appropriate level of expertise to confirm that it is of an acceptable scientific standard.

# Peer review: Referee response for version 1

**DOI:** 10.5256/f1000research.4486.r6142

**Published:** 2014-09-16

**Authors:** Nitika Pant Pai, Jana Daher

**Affiliations:** 1 Division of Clinical Epidemiology, Department of Medicine, Royal Victoria Hospital, McGill University Health Centre, Montreal, QC, Canada; 2 Division of Clinical Epidemiology, Department of Medicine, Royal Victoria Hospital, McGill University Health Centre, Montreal, QC, Canada

**Overall:**

- This manuscript must be indexed subject to revisions/clarifications that are highlighted in specific sections mentioned below.

 

**Strengths:**

- The strengths of the study lay in its design, recruitment of displaced, vulnerable populations, and evaluation of rapid testing interventions (antenatal screening for HIV and Syphilis) with downstream implications for mother-to-infant transmission. 
- Data on this population are sparse. Little is known about effectiveness of screening in migrant populations and actions taken to link them to care and prevention.

**Limitations:**

- A convenience sample is enrolled - leading to a biased sample of a migrant population whose primary study base is ill-defined. This limits generalizability of their findings. 
- Confounding: a discussion is needed - especially because a multivariate analyses was not attempted. 
- A small sample, and fewer infections detected in the small sample, limits a multivariate analysis. 
- Two groups are compared with a null finding.
- A clear hypotheses, a clear primary objective and a clean sample size calculation based on that is needed.
- Implication section for policy and practice is missing.

Detailed suggestions are provided below:

**Title:**

- The title specifies the studied population, the location and the correct study design.
- A suggested alternative title could be: “Audit of the antenatal screening for syphilis and HIV in migrant and refugee women on the Thai-Myanmar border: a descriptive study” for the following reasons:
  ​
  - The HIV and syphilis prevalence estimates obtained when screening the sampled population may not be a true reflection of the prevalence among the underlying source population (refer to the limitation in the discussion section below).
  - The rates of infections are not the only focus of the study. The study also explores counselling challenges following screening and some consequential considerations for future testing strategies; however these are not covered in the title.

**Abstract:**

- The abstract provides informative and balanced summary of the study.
- There is a discrepancy between the aim presented in the abstract and the one presented in the introduction. The aim of auditing the first year of the syphilis routine screening is mentioned in the abstract but not the one of reassessing the trends in HIV rates.

**Introduction:**

- The scientific background is provided and the rationale for the investigation was well explained: the 2 studied STIs present major health risks on maternal and child health (it would be useful to add the health risk on the woman and not only on the pregnancy outcomes). The pregnant woman is especially vulnerable to infection when displaced (low income was mentioned as a risk factor – along with low education and lack of access to health services – and it would be useful to explain the correlation). There is a large displaced population between Thailand and Myanmar and data on prevalence of infections among this population is scarce.
- The objective of the study is stated. The aim of auditing a screening program is quite ambitious (many of its facets are discussed which may be overwhelming to the reader).

**Methods:**

- The study design is overall well presented (including study site, data collection and laboratory testing, treatment plan and adequate statistical analysis plan).
- Regarding the study population, the eligibility criteria and the sample size considerations (sample should be large enough to reflect important variations) are not discussed.
- Training and expertise of the people executing the screening tests is not mentioned. Authors should discuss the comparability of the testing methods in the three SMRU clinics included in the study and the quality assurance and control measures applied.

**Results:**

- The results section presents and adequately summarizes all relevant characteristics of the study participants (Table 1). However, it would be helpful to define the number of marriages that a woman has as the number of remarriages (which is explained in the discussion). Literacy should also be defined. Additionally, 2 MLA and 14 WPA patients are not segregated into the different ethnic groups and are therefore missing from this part of the table; this mismatch can be detected when computing the total number of participants from each of these two sites. Finally, it is advised to keep one of the two significance levels presented below the table (P<005 or P<0.001).
- The results on prevalence rates of syphilis and HIV are properly presented in Table 2. However, the data from other studies on prevalence rates in Myanmar and Thailand are not very informative given the fact that confidence intervals are not provided.
- The reasons for non-participation and missing data are provided for each of those who did not complete the screening. However, the authors should consider providing an explanation for the one instance where a partner was treated for syphilis without being tested first.
- A multivariate analysis would have been more informative than a univariate analysis in order to get a clearer understanding of the risk factors associated with contracting syphilis and HIV. But, as the authors mention, this type of analysis is not possible when having a small number of cases. The authors should consider adding the type of analysis in the title of Table 3. Also, the confidence interval of the odds ratio for syphilis in the migrant group (1.182 – 6.921 instead of 1.182- 69.214) A wide confidence interval could be due to small number of cases, and sampling variability issues - these could be acknowledged.

**Discussion:**

- The discussion summarises the key results. But the conclusion on the HIV rates does not accurately encapsulate what is presented in the results section. It is advised to state that no statistically significant difference in HIV rates is observed between the refugees and the combined migrant population attending both MKT and WPA sites (since it is mentioned in the results section that there is a statistically significant difference in HIV rates between MLA and WPA).
- The discussion is contextualized in relation to the studied population. The authors present sound explanations and interpretations of the results based on a good understanding of the cultural traits and traditions of the studied population.
- The authors discuss the clinical applicability of the study findings. It would be useful to further clarify the ethical difficulties faced and the challenges created during post-test counselling when a false positive syphilis result is undetected. For example, the authors can explain that a positive syphilis screening test does not necessarily confirm syphilis when titres are low and that yaws – which is not sexually transmitted – may instead be suspected. This inability to determine the true cause of the positive result can lead to serious consequences when counselling a patient with low titres levels – as it may increase her risk of abuse and abandonment. It can also be specified that that yaws are transmitted through skin-to-skin contact and promoted by overcrowding and poor hygiene (which is very likely the case in the study population).
- The authors discuss limitations of the study. However, the convenience sampling of the study population is a limitation that is not mentioned in the discussion. There is a risk of selection bias since the study population consists of regular attendees of the SMRU antenatal clinics who may be very different from those who are not. For example, regular attendees of the clinic may be more health conscious – engage in less risky behaviors – and may have been more exposed to health recommendations and counselling than those who miss antenatal care visits.
- The generalizability of results (external validity) to the whole population of migrants and refugees in the studied location is difficult to establish given the risk of selection bias. 

In conclusion, the study is scientifically sound however some editing is advised. It presents interesting data on HIV and syphilis prevalence for regular antenatal clinic attendees coming from a disadvantaged and understudied population of migrant and refugee at the Thai- Myanmar border.

It provides valuable ethical considerations and highlights a caveat pertaining to the current

syphilis testing strategy and post-test counselling.

This review could help public health officials better understand the situation in regard to HIV

and syphilis among the studied population in order to revise the routine screening program.

We have read this submission. We believe that we have an appropriate level of expertise to confirm that it is of an acceptable scientific standard, however we have significant reservations, as outlined above.

# Response

**Published:** 2015-05-05

**Authors:** Rose McGready

**Affiliations:** Tropical Medicine Division, Shoklo Malaria Research Unit, Thailand

We appreciate and would like to sincerely thank the significant effort of both reviewers in regards to this manuscript. We apologize for the tardiness of the response. We have provided a point by point response to the comments below.

**Overall:**

- **This manuscript must be indexed subject to revisions/clarifications that are highlighted in specific sections mentioned below.**

** Strengths:**

- **The strengths of the study lay in its design, recruitment of displaced, vulnerable populations, and evaluation of rapid testing interventions (antenatal screening for HIV and Syphilis) with downstream implications for mother-to-infant transmission. **
** **
- **Data on this population are sparse. Little is known about effectiveness of screening in migrant populations and actions taken to link them to care and prevention.**

**Limitations:**

- **A convenience sample is enrolled - leading to a biased sample of a migrant population whose primary study base is ill-defined. This limits generalizability of their findings. **

Thank you – we need to clarify. This is not a convenience sample but the whole population of pregnant women in well attended antenatal clinics. This point is similar to point 2 raised by reviewer one about the population. The methods (2 ^nd^ paragraph) has been modified to better describe this population.

- **Confounding: a discussion is needed - especially because a multivariate analyses was not attempted.**

The original submissions did a univariate analysis (Table 3) and in the results a sentence was added “Further modelling was not done due to the low number of seropositive cases precluding meaningful conclusions.” At this reviewer’s request a multivariate analysis has been added here (next page) and as you can see the confidence intervals of the AOR are very large, which means the level of uncertainty are so high that we cannot confidently interpret the result. If the reviewer insists we can include the multivariate analysis in the main body of the paper with the appropriate changes to the methods section as well (included here).

Methods, statistical analysis: Factors associated with a diagnosis of syphilis or a diagnosis of HIV, were evaluated by univariate analysis; two logistic regression models were created using “syphilis (yes/no)” and “HIV (yes/no)” as dependent variables. All factors with a P < 0.10 in univariate analysis were entered in their respective stepwise forward logistic regression model, and were included in the relevant tables. Adjusted odds ratios (AOR) were given with their 95% confidence interval.

[see table 3 in new version - resubmission]

 

- **A small sample, and fewer infections detected in the small sample, limits a multivariate analysis. **

We have acknowledged this. The main reason to write this manuscript is not to explore risk factors but to question the prevalence at which syphilis testing is not viable for resource constrained settings.

- **Two groups are compared with a null finding.**

Yes – the incidence if HIV and syphilis is very low in both populations which is what we are trying to illustrate

- **A clear hypotheses, a clear primary objective and a clean sample size calculation based on that is needed.**

This is a non-selected inclusive cohort of all consecutively registered pregnant women so no sample size was provided. This has been clarified, 2 ^nd^ paragraphs in the methods.

- **Implication section for policy and practice is missing.**

These implications are clarified in the paragraph starting at line 293.

**Detailed suggestions are provided below:**

**Title:**

- **The title specifies the studied population, the location and the correct study design.**
- **A suggested alternative title could be: “Audit of the antenatal screening for syphilis and HIV in migrant and refugee women on the Thai-Myanmar border: a descriptive study” for the following reasons**:
  - **The HIV and syphilis prevalence estimates obtained when screening the sampled population may not be a true reflection of the prevalence among the underlying source population (refer to the limitation in the discussion section below). **
  - **The rates of infections are not the only focus of the study. The study also explores counselling challenges following screening and some consequential considerations for future testing strategies; however these are not covered in the title.**

We have amended the title in line with what has been suggested although we are confident that we have good coverage of the population. We agree with the second point. 

**Abstract:**

- **The abstract provides informative and balanced summary of the study.**
** There is a discrepancy between the aim presented in the abstract and the one presented in the introduction. The aim of auditing the first year of the syphilis routine screening is mentioned in the abstract but not the one of reassessing the trends in HIV rates.**

Thank you for this comment and the aims are now aligned.

**Introduction:**

- **The scientific background is provided and the rationale for the investigation was well explained: the 2 studied STIs present major health risks on maternal and child health (it would be useful to add the health risk on the woman and not only on the pregnancy outcomes). **

We agree and a sentence added to the end of the first paragraph of the introduction.

**The pregnant woman is especially vulnerable to infection when displaced (low income was mentioned as a risk factor – along with low education and lack of access to health services – and it would be useful to explain the correlation). There is a large displaced population between Thailand and Myanmar and data on prevalence of infections among this population is scarce.**

Thank and this has been amended in the introduction (see end of the 2 ^nd^ paragraph).

- **The objective of the study is stated. The aim of auditing a screening program is quite ambitious (many of its facets are discussed which may be overwhelming to the reader).**

Given the challenges we encountered, we found that such an audit (ambitious and complex as it is) is necessary to shed light on the issues faced in this context.  It is our hope that the overwhelming experience of the reader will be lessened by the changes we have made in response to the general reviewer comments.  No specific changes made to change the objective.

**Methods:**

- **The study design is overall well presented (including study site, data collection and laboratory testing, treatment plan and adequate statistical analysis plan).**

OK

 

- **Regarding the study population, the eligibility criteria and the sample size considerations (sample should be large enough to reflect important variations) are not discussed**

Thank you.  The sample is exhaustive and the study population is more clearly defined now in the methods section.

- **Training and expertise of the people executing the screening tests is not mentioned. Authors should discuss the comparability of the testing methods in the three SMRU clinics included in the study and the quality assurance and control measures applied.**

Thank you for this and the information has been added to the first paragraph of laboratory sampling.

**Results:**

- **The results section presents and adequately summarizes all relevant characteristics of the study participants (Table 1). However, it would be helpful to define the number of marriages that a woman has as the number of remarriages (which is explained in the discussion).** 

Amended

**Literacy should also be defined.**

Amended: this was self-reported.

**Additionally, 2 MLA and 14 WPA patients are not segregated into the different ethnic groups and are therefore missing from this part of the table; this mismatch can be detected when computing the total number of participants from each of these two sites.**

Thank you for detecting this. It is an error and the numbers in the manuscript has been carefully reviewed in line with this finding.

**Finally, it is advised to keep one of the two significance levels presented below the table (P<005 or P<0.001).**

Amended: only P<0.05 has been maintained.

- **The results on prevalence rates of syphilis and HIV are properly presented in Table 2. However, the data from other studies on prevalence rates in Myanmar and Thailand are not very informative given the fact that confidence intervals are not provided**

We think the comparative data compiled in one table is helpful to understand the situation of a population that is rarely provided with any type of sero-surveillance. We agree a better data source that provides confidence intervals would be useful but we are unable to find exact numbers for Thailand. We have expanded the paragraph in the results further and provided clearer reference material; including confidence intervals for the Myanmar data.

**The reasons for non-participation and missing data are provided for each of those who did not complete the screening. However, the authors should consider providing an explanation for the one instance where a partner was treated for syphilis without being tested first.**

High risk case and this has been added to the results section.

- **A multivariate analysis would have been more informative than a univariate analysis in order to get a clearer understanding of the risk factors associated with contracting syphilis and HIV. But, as the authors mention, this type of analysis is not possible when having a small number of cases. The authors should consider adding the type of analysis in the title of Table 3. Also, the confidence interval of the odds ratio for syphilis in the migrant group (1.182 – 6.921 instead of 1.182- 69.214) A wide confidence interval could be due to small number of cases, and sampling variability issues - these could be acknowledged.**

Analysis of risk factors is not the main objective of this manuscript, and the limitation of such an analysis due to low positivity is acknowledged in the original submission. We have clarified in the laboratory sampling section that sampling variability is minimized by regular quality control and standardization, and this is not a significant contributor to the wide confidence interval, unlike the small number of positive cases. The comment about the confidence interval of the odds ratio for syphilis is a little unclear to us, but the correct CI is as included in the table (1.182-69.214) i.e. it really is 69 and no 6.9.

**Discussion:**

- **The discussion summarises the key results. But the conclusion on the HIV rates does not accurately encapsulate what is presented in the results section. It is advised to state that no statistically significant difference in HIV rates is observed between the refugees and the combined migrant population attending both MKT and WPA sites (since it is mentioned in the results section that there is a statistically significant difference in HIV rates between MLA and WPA).**

Thank you for this suggestion and it has been amended as suggested.

- **The discussion is contextualized in relation to the studied population. The authors present sound explanations and interpretations of the results based on a good understanding of the cultural traits and traditions of the studied population.**
- **The authors discuss the clinical applicability of the study findings. It would be useful to further clarify the ethical difficulties faced and the challenges created during post-test counselling when a false positive syphilis result is undetected. **

**For example, the authors can explain that a positive syphilis screening test does not necessarily confirm syphilis when titres are low and that yaws – which is not sexually transmitted – may instead be suspected. This inability to determine the true cause of the positive result can lead to serious consequences when counselling a patient with low titres levels – as it may increase her risk of abuse and abandonment. It can also be specified that that yaws are transmitted through skin-to-skin contact and promoted by overcrowding and poor hygiene (which is very likely the case in the study population).**

 We have amended the text as suggested.

- **The authors discuss limitations of the study. However, the convenience sampling of the study population is a limitation that is not mentioned in the discussion. **

**There is a risk of selection bias since the study population consists of regular attendees of the SMRU antenatal clinics who may be very different from those who are not. For example, regular attendees of the clinic may be more health conscious – engage in less risky behaviors – and may have been more exposed to health recommendations and counselling than those who miss antenatal care visits.**

Sampling was exhaustive, it was not a convenience sample and we included women who came for only one ANC visit or arrived at the clinic for the first time while in labor.

- **The generalizability of results (external validity) to the whole population of migrants and refugees in the studied location is difficult to establish given the risk of selection bias. **

The sampling was exhaustive amongst consecutively enrolled pregnant women. The data is similar to previous surveys and consistent with other reports from the area suggesting the risk of selection bias is low. This has been better clarified in relation to the methods (the study population) and relation to other data sources included in the first paragraph of the discussion.

**In conclusion, the study is scientifically sound however some editing is advised. It presents interesting data on HIV and syphilis prevalence for regular antenatal clinic attendees coming from a disadvantaged and understudied population of migrant and refugee at the Thai- Myanmar border.**

**It provides valuable ethical considerations and highlights a caveat pertaining to the current syphilis testing strategy and post-test counselling.**

**This review could help public health officials better understand the situation in regard to HIV**

**and syphilis among the studied population in order to revise the routine screening program.**

# Peer review: Referee response for version 1

**DOI:** 10.5256/f1000research.4486.r5072

**Published:** 2014-06-27

**Authors:** Matthew Chico

**Affiliations:** 1 Department of Disease Control, London School of Hygiene & Tropical Medicine, London, UK

**Overall comment:**

This is a well-written paper on an important topic, for which there is limited data: What is the prevalence below which routine screening of all pregnant women for HIV and, in particular, syphilis is no longer advisable? 

**Specific comments:**

1. Syphilis is far more deleterious to birth outcomes than other bacterial sexually transmitted infections and, therefore, the potential risks associated with missed diagnoses should be made in clearer in the paper.  Among unscreened pregnant women in Tanzania, 51% of stillbirths, 24% of preterm live births, and 17% of all adverse pregnancy outcomes were attributable to maternal syphilis ^1^. The authors should consider citing the comprehensive summary of the effect of curable STIs on birth outcomes by Chico *et al.* (Table 1. Effect of curable STIs/RTIs on pregnancy outcomes) ^2^.
2. Clearly the rates of HIV and syphilis were low among the refugees and migrants tested along the Thai-Myanmar border.  However, it is not clear in the manuscript (1) the proportion of pregnant women in these two populations who seek antenatal care services, and (2) the prevalence of syphilis and HIV in the two populations who do not seek ANC services.  Are the pregnant women attending these health facilities healthier than the overall population?  Pregnant women are encouraged to attend ANC clinics as frequently as every fortnight.  This makes it more difficult to generalise results beyond the study population.
3. The refugee and migrant populations in this setting are unique for cultural reasons described in the paper. There should be some discussion about other contexts in which women are at elevated risk of HIV and syphilis infection, and that women may enter into transient marital relationships that are based in personal security, but expose them to other risks.
4. There should be some discussion about the use of rapid point of care (POC) tests for syphilis, in place of VDRL and THPA assays.  POC tests for syphilis simplify the screening and treatment procedures.  The World Health Organization currently recommends the use of POC tests for syphilis in the antenatal care setting ^3^. Using POC tests will expedite case finding and treatment because results will be available during the same consultation. Although published results are not yet available, a combination HIV and syphilis POC test has recently been validated.
5. The paper would benefit from a table that delineates the demographic and birth-outcome data for the 14 women who were found to have syphilis. The risk profile in Table 3 suggests that (1) migrant women, (2) who have been married more than once, (3) who are above the age of 30, (4) whose parents are not Karen, and (5) who have lived at their present address less than six months, are most likely to have a syphilis infection. Considering these five risk factors, how many women had just one, or two, or three, or four, or all five?

I have read this submission. I believe that I have an appropriate level of expertise to confirm that it is of an acceptable scientific standard.

## References

[1] Watson-JonesDChanhaluchaJGumodokaBWeissHRusizokaMNdekiLWhitehouseABaliraRToddJNgelejaDRossDBuvéAHayesRMabeyD: Syphilis in pregnancy in Tanzania. I. Impact of maternal syphilis on outcome of pregnancy. *J Infect Dis*.2002;186(7) : 123 940-947 10.1086/342952 Reference source 12232834

[2] ChicoRMHackBBNewportMJNgulubeEChandramohanD: On the pathway to better birth outcomes? A systematic review of azithromycin and curable sexually transmitted infections. *Expert Rev Anti Infect Ther*.2013;11(12) : 123 1303-1332 10.1586/14787210.2013.851601 Reference source 24191955PMC3906303

[3] World Health Organization: The use of rapid syphilis tests: Geneva:WHO.2006; Reference source

# Response

**Published:** 2015-05-05

**Authors:** Rose McGready

**Affiliations:** Tropical Medicine Division, Shoklo Malaria Research Unit, Thailand

We appreciate and would like to sincerely thank the significant effort of both reviewers in regards to this manuscript. We apologize for the tardiness of the response. We have provided a point by point response to the comments below.

**This is a well-written paper on an important topic, for which there is limited data: What is the prevalence below which routine screening of all pregnant women for HIV and, in particular, syphilis is no longer advisable?**

**Specific comments:**

**1.Syphilis is far more deleterious to birth outcomes than other bacterial sexually transmitted infections and, therefore, the potential risks associated with missed diagnoses should be made in clearer in the paper.  Among unscreened pregnant women in Tanzania, 51% of stillbirths, 24% of preterm live births, and 17% of all adverse pregnancy outcomes were attributable to maternal syphilis** **^1^** **. The authors should consider citing the comprehensive summary of the effect of curable STIs on birth outcomes by Chico *et al.* (Table 1. Effect of curable STIs/RTIs on pregnancy outcomes)** **^2^** **.**

We agree this is important and have amended the text as suggested in the 2 ^nd^ paragraph of the introduction. 

**2.Clearly the rates of HIV and syphilis were low among the refugees and migrants tested along the Thai-Myanmar border.  However, it is not clear in the manuscript (1) the proportion of pregnant women in these two populations who seek antenatal care services, and (2) the prevalence of syphilis and HIV in the two populations who do not seek ANC services.  Are the pregnant women attending these health facilities healthier than the overall population?  Pregnant women are encouraged to attend ANC clinics as frequently as every fortnight.  This makes it more difficult to generalise results beyond the study population.**

Thank you for pointing this out and this information has been added to the methods section.

1. We estimate the proportion at 90% - see 2 ^nd^ paragraph of the methods section.
2. The “prevalence of syphilis and HIV in the two populations who do not seek ANC services” is impossible to answer. Data of HIV rates from the border wide camp Health information system suggest similarly low prevalence and this has been added to the discussion first paragraph.

The ANC has always encouraged fortnightly voluntary visits (and care during childbirth in the clinic) and 4 or less antenatal visits in this setting has been associated with an increased risk of maternal death: AOR 2.50 (95%CI 1.41–4.43) ( doi: 10.1371/journal.pone.0040244) and this detail has been been added to the methods first paragraph. 

**3.The refugee and migrant populations in this setting are unique for cultural reasons described in the paper. There should be some discussion about other contexts in which women are at elevated risk of HIV and syphilis infection, and that women may enter into transient marital relationships that are based in personal security, but expose them to other risks.**

This has been added into the discussion towards the end of the first paragraph in the conclusions.

**4.There should be some discussion about the use of rapid point of care (POC) tests for syphilis, in place of VDRL and THPA assays.  POC tests for syphilis simplify the screening and treatment procedures.  The World Health Organization currently recommends the use of POC tests for syphilis in the antenatal care setting** **^3^** **. Using POC tests will expedite case finding and treatment because results will be available during the same consultation. Although published results are not yet available, a combination HIV and syphilis POC test has recently been validated.**

We have not got experience with POC for syphilis. Our experience with POC for HIV (higher positivity rate than syphilis) has not been good as explained in the results – nearly ½ were false positive. As the incidence goes down the false positive rate goes up and this is one of the reasons we have not moved to POC tests for syphilis in this population. Currently the turn around time with the local Thailand hospital where the POC positive results are sent for confirmation and the distance involved is a minimum of 3 days (usually a week). This is very stressful for women. This has been added to the discussion. We are not against POC tests and use malaria POC testing frequently. POC tests have limitations in their own right and the limitations and advantages need to be determined locally.

On another note we have also found that hepatitis B POC tests also present us with a similar problem but to a lesser degree (the incidence of Hepatitis B in adults in this area approaches 10%). Approximately 10% of Hepatitis B Surface Ag POC positive tests turn out to be incorrect. This is not a problem when the point is to screen for healthy donors but it is a problem when you are telling an individual they have a chronic disease.

We would be delighted to be a site for testing sensitivity and specificity of such tests.

**5.The paper would benefit from a table that delineates the demographic and birth-outcome data for the 14 women who were found to have syphilis. The risk profile in Table 3 suggests that (1) migrant women, (2) who have been married more than once, (3) who are above the age of 30, (4) whose parents are not Karen, and (5) who have lived at their present address less than six months, are most likely to have a syphilis infection. Considering these five risk factors, how many women had just one, or two, or three, or four, or all five?**

We appreciate this suggestion as a method to try to focus on at risk women. We have looked at this in 2 ways: scoring the number of risk factors a woman has (below)

We appreciate this suggestion as a method to try to focus on at risk women. We have looked at this in 2 ways: scoring the number of risk factors a woman has (below)

Score

Syphilis n=14

No syphilis n=3578

0

0 (0)

646 (100%)

1

2* (0.2%)

1082 (99.8%)

2

0 (0)

1032 (100%)

3

4 (0.7%)

1. 99.3%)

4

5 (2.3%)

216 (97.7%)

5

3 (10.0%)

27 (90.0%)

*Scored: one woman scored 1 because of ‘migrant’ status; and one woman scored 1 because of age *≥ 30 y.*

A risk factor score of ≥  3 includes  1.44% (12/831) and a  score <3 includes 0.07% ( 2/2762).

And trying to identify if a pattern of risk factors is common (below)

 Finally as suggested by the 2 ^nd^ reviewer we have run a multivariate analysis which can be found at the request of the 2 ^nd^ reviewer (see comments). The main message of the manuscript is not to identify risk factors (which also does not seem practical given the low number of positive case).

**References**

1. Watson-Jones D, Chanhalucha J, Gumodoka B, Weiss H, Rusizoka M, Ndeki L, Whitehouse A, Balira R, Todd J, Ngeleja D, Ross D, Buvé A, Hayes R, Mabey D: Syphilis in pregnancy in Tanzania. I. Impact of maternal syphilis on outcome of pregnancy. *J Infect Dis*. 2002; **186** (7): 940-947 PubMed Abstract | Publisher Full Text | Reference Source

2. Chico RM, Hack BB, Newport MJ, Ngulube E, Chandramohan D: On the pathway to better birth outcomes? A systematic review of azithromycin and curable sexually transmitted infections. *Expert Rev Anti Infect Ther*. 2013; **11** (12): 1303-1332 PubMed Abstract | Free Full Text | Publisher Full Text | Reference Source

3. World Health Organization: The use of rapid syphilis tests: Geneva:WHO. 2006. Reference Source
